# Inhibition of endothelial nitric oxide synthase in cholangiocarcinoma cell lines – a new strategy for therapy

**DOI:** 10.1002/2211-5463.12388

**Published:** 2018-03-02

**Authors:** Manida Suksawat, Anchalee Techasen, Nisana Namwat, Thianrut Boonsong, Attapol Titapun, Piti Ungarreevittaya, Puangrat Yongvanit, Watcharin Loilome

**Affiliations:** ^1^ Department of Biochemistry Faculty of Medicine Khon Kaen University Thailand; ^2^ Cholangiocarcinoma Research Institute Khon Kaen University Thailand; ^3^ Faculty of Associated Medical Science Khon Kaen University Thailand; ^4^ Cholangiocarcinoma Screening and Care Program (CASCAP) Khon Kaen University Thailand; ^5^ Department of Surgery Faculty of Medicine Khon Kaen University Thailand; ^6^ Department of Pathology Faculty of Medicine Khon Kaen University Thailand

**Keywords:** cholangiocarcinoma, eNOS, invasion, metastasis, migration

## Abstract

The isoform of nitric oxide synthase (NOS) found in endothelial cells (eNOS) plays a crucial role in vasodilation. We recently reported the activation of eNOS in cholangiocarcinoma (CCA) tissues and cell lines. Moreover, we also reported that the abundance of eNOS and phosphorylated eNOS (p‐eNOS), as well as its upstream regulator proteins, is significantly associated with the metastatic status of CCA patients. However, the function of eNOS in CCA progression has not been addressed. Therefore, the present study aimed to investigate the function of eNOS involved in the migration and invasion ability of CCA cell lines. The results reveal that eNOS activation significantly increases migration and invasion ability of CCA cells *via* the up‐regulation of phosphorylated vasodilator‐stimulated protein (p‐VASP). A combination treatment with recombinant human vascular endothelial growth factor C and eNOS inhibitor (*N* ^ω^‐nitro‐l‐arginine methyl ester hydrochloride) resulted in the down‐regulation of p‐VASP, as well as a decreased migration and invasion ability of the CCA cell line. Thus, this work suggests that eNOS can serve as an attractive target to inhibit the progression of CCA.

**Abbreviations**
CCA: cholangiocarcinoma
eNOS: endothelial nitric oxide synthase
IL‐18: interleukin‐18
iNOS: inducible nitric oxide synthase
l‐NAME: *N* ^ω^‐nitro‐l‐arginine methyl ester hydrochloride
nNOS: neuronal nitric oxide synthase
NO: nitric oxide
NOS: nitric oxide synthase
Ov: *Opisthorchis viverrini*
PI3K: phosphoinositide‐3‐kinase
rhVEGF‐C: recombinant human vascular endothelial growth factor C
VASP: vasodilator‐stimulated protein
VEGF: vascular endothelial growth factor
VEGFR: vascular endothelial growth factor receptor

Cholangiocarcinoma (CCA) is an invasive cancer that originates from the bile duct epithelium. It has a high incidence and has been recognized as a major public health problem in the northeastern part of Thailand 1. The development and pathogenesis of CCA is associated with liver fluke (*Opisthorchis viverrini*; Ov) infection resulting in chronic inflammation of bile duct. This is recognized as the major risk factor for CCA development in this region 2, 3. In addition, the alteration of genes and proteins involved in the kinase pathway can promote CCA cell growth and migration 4, 5, 6, 7, 8, 9, 10.

We previously found that multiple protein kinases, including membrane receptor tyrosine kinases and cytoplasmic kinases, are over‐activated in CCA. Among the activated proteins, endothelial nitric oxide synthase (eNOS) phosphorylated at serine 1177 (p‐eNOS (Ser1177)) was found to be overactivated in both CCA cell lines and CCA tissues. Beside eNOS, vascular endothelial growth factor receptor (VEGFR) 3 was also found activated in CCA cell lines and tissues 4. VGFR3 has vascular endothelial growth factor (VEGF) C and D as its specific ligands. However, based on our review the axis of VEGF‐C–VEGFR3 is more strongly associated with eNOS and cancer progression than is VEGF‐D. In addition, we reported the association of eNOS with VEGF‐C and VEGFR3, which can act as upstream regulators of eNOS. We also demonstrated that the abundance of eNOS, p‐eNOS (Ser1177) and its upstream regulator proteins, VEGF‐C and VEGFR, was associated with the metastatic status of CCA patients. Interestingly, the co‐high‐expression of eNOS/p‐eNOS and its upstream regulators also significantly correlated with metastasis in CCA patients 11. These results convinced us to focus on the role of eNOS in the migration and invasion ability in an *in vitro* CCA model.

The nitric oxide (NO)‐generating enzyme nitric oxide synthase (NOS) has three isoforms: neuronal (nNOS), inducible (iNOS) and endothelial (eNOS) 12. Normally, eNOS is mainly found in endothelial cells where it plays an important role in vascular relaxation 13. eNOS also plays roles in pathological processes, including cancer development 14, 15, 16. It has been detected in various types of cancer 17, 18, where it is involved in carcinogenesis, including cell proliferation in oral squamous cancer cell lines 19 and antiapoptosis in prostate cancer cells 18. Moreover, eNOS is involved in angiogenic processes in gastric cancer 20 and promotes invasion and metastasis in mammary cancer cells 21. eNOS is strongly regulated by VEGF through the phosphoinositide‐3‐kinase (PI3K)–Akt pathway 22 resulting in p‐eNOS (Ser1177) and an increase in NO production 23. Interestingly, a high level of activation of the VEGFR3 and PI3K–Akt pathway has been found also in CCA 4, 10. Thus, the inhibition of eNOS may act as a therapeutic strategy to inhibit cancer progression, potentially *via* the use of *N* ^ω^‐nitro‐l‐arginine methyl ester hydrochloride (l‐NAME), an l‐arginine analogue that is an eNOS inhibitor. l‐NAME is approximately 10 times more specific for eNOS than for iNOS, and is widely used to inhibit eNOS activity in many types of cancer 24.

In the present study we explored the function of eNOS in CCA migration and invasion. The induction of eNOS activation was performed by human recombinant VEGF‐C (rhVEGF‐C) in combination with l‐NAME, an eNOS inhibitor. In addition, the molecular mechanism by which eNOS regulates CCA cell migration and invasion was demonstrated.

## Materials and methods

### Human CCA cell lines and cell culture

Human CCA cell line KKU‐213 was purchase from the JCRB cell bank. The cell line was cultured in Dulbecco's modified Eagle's medium (DMEM) (Thermo Fisher Scientific, Waltham, MA, USA) supplemented with 10% fetal bovine serum, 44 mm NaHCO_3_, penicillin (100 units·mL^−1^) and streptomycin (100 mg·mL^−1^) (Thermo Fisher Scientific), and the cell line was grown in a humidified atmosphere at 37 °C containing 5% CO_2_.

### Antibodies

Primary antibodies were used in this study, including anti‐eNOS, which was purchased from BD Bioscience (San Jose, CA, USA), and p‐eNOS purchased from Abcam (Cambridge, MA, USA) for immunolabeling. The antibodies used for immunoblotting were as follows: anti‐PI3K, AKT, p‐Akt (Ser473), p‐VASP (Ser239) and MMP9 were purchased from Cell Signaling Technology (Danvers, MA, USA), and anti‐β‐actin antibody from Sigma‐Aldrich (St Louis, MO, USA).

### Recombinant human VEGF‐C and eNOS inhibitor

The recombinant human VEGF‐C (rhVEGF‐C) cys156ser, which is specific for VEGFR3, was purchased from R&D system (Minneapolis, MN, USA). rhVEGF‐C was reconstituted in sterile PBS at a stock concentration of 10^5^ ng·mL^−1^ and stored at −20 °C until used. The inhibitor of eNOS, l‐NAME, was purchased from Sigma‐Aldrich.

### Immunolabeling localization of eNOS and p‐eNOS

The CCA cell line was plated into eight‐well slide chambers for 24 h and treated with 100 ng·mL^−1^ of rhVEGF‐C and the combination of 100 ng·mL^−1^ rhVEGF‐C and different concentrations (1, 10 and 100 μm) of l‐NAME for 24 h. The cells were then fixed using 4% paraformaldehyde for 30 min followed by non‐specific blocking in 3% BSA for 1 h at room temperature. They were then incubated with primary antibody to eNOS or p‐eNOS (dilution 1 : 200 for all) at 4 °C overnight. After washing, the cells were incubated with Alexa Fluor 555‐labeled goat anti‐mouse IgG and Alexa Fluor 488‐labeled goat anti‐rabbit IgG for eNOS and p‐eNOS, respectively (Thermo Fisher Scientific). Finally, nucleus staining with 4′,6‐diamidino‐2‐phenylindole was performed.

The results were analyzed using a confocal scanning microscope (×20 and ×63) (Zeiss LSM 800, Carl Zeiss, Oberkochen, Germany). After detection, the quantification of immunofluorescence density was performed with ImageJ and calculated following the formula:

Corrected total cell fluorescence = Integrated density – (Area of selected cell × Mean fluorescence of background readings). This quantification method followed from previous publications 25, 26.

### Western blot analysis

Cells were treated with 100 ng·mL^−1^ rhVEGF‐C and the combination of 100 ng·mL^−1^ rhVEGF‐C and different concentrations of l‐NAME (1, 10 and 100 μm) for 24 h. Cell lysates were electrophoresed and transferred to a poly(vinylidene) difluoride membrane (Millipore, Billerica, MA, USA). This was blocked with 5% skim milk in Tris‐buffered saline (TBS) at room temperature for 1 h and incubated with primary antibody at 4 °C overnight. After that, it was rinsed with TBS containing 0.1% polyoxyethylenesorbitan monolaurate (Tween‐20; TBST) followed by incubation with horseradish peroxidase‐conjugated secondary antibody from Santa Cruz Biotechnology (Dallas, TX, USA) at room temperature for 1 h. The membranes were rinsed with TBST and exposed to ECL Prime Western Blotting Detection System (GE Healthcare Bio‐Sciences, Little Chalfont, UK). The immunoblot and intensity were analyzed with the ImageQuant™ analysis system (GE Healthcare Bio‐Sciences). Human β‐actin served as the loading control.

### Migration assay

A cell migration assay was performed using a Boyden chamber transwell consisting of a membrane filter insert in 24‐well plates, 8 μm pore size (Corning, New York, USA). The KKU‐213 (4 × 10^4^ cells) cell line was pretreated with 100 ng·mL^−1^ of rhVEGF‐C and the combination of 100 ng·mL^−1^ rhVEGF‐C and different concentrations of l‐NAME (1, 10 and 100 μm) for 30 min before seeding into the upper chamber with serum free medium. The lower chamber contained complete medium and was incubated for 24 h. After 24 h, non‐migrating cells in the upper chamber were removed. Migrating cells that attached at the underside of the filter were fixed with ethanol for 1 h and stained overnight with hematoxylin. Quantification of the migrating cells was performed by counting under a light microscope (×20 magnification). The experiments were carried out in duplicate and two independent experiments were repeated.

### Invasion assay

A Boyden chamber with an insert filter coated with Matrigel, 8‐μm pore size (Corning), was used for the invasion assay. The assay kit was reconstituted by placing serum‐free medium into the upper chamber and complete medium into the lower chamber for approximately 1 h. At the same time, KKU‐213 (4 × 10^4^) cells were pretreated with 100 ng·mL^−1^ of rhVEGF‐C and the combination of 100 ng·mL^−1^ rhVEGF‐C and different concentrations of l‐NAME (1, 10 and 100 μm) for 30 min. Before seeding the pretreated cells, serum‐free medium was removed and replaced with the pretreated cells in the upper chamber. After 24 h, non‐invading cells that were found in the upper filter were removed. Invading cells that attached under the filter were fixed with ethanol for 1 h followed by staining overnight with hematoxylin. The quantification of invading cells was performed by counting under a light microscope (×20 magnification). The experiments were carried out in duplicate and two independent experiments were repeated.

### Statistical analysis

Statistical analyses were performed with SPSS Statistics version 17 (SPSS Inc., Chicago, IL, USA). The difference between groups was expressed as a mean ± SD and analyzed by Student's *t* test. A *P‐*value less than 0.05 was considered statistically significant.

## Results

### rhVEGF‐C induced expression level of p‐eNOS

**Figure 1:**
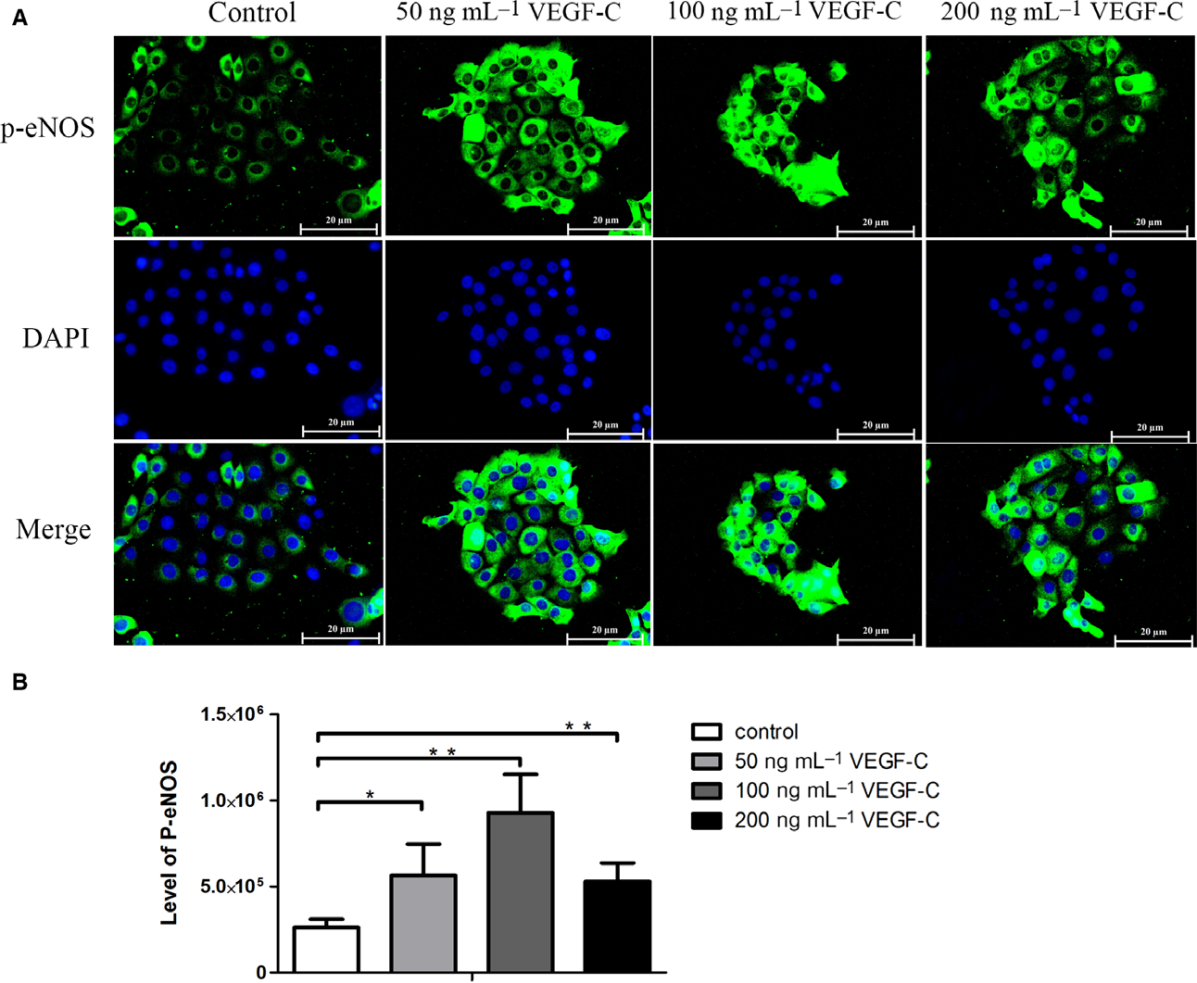
(A) Level of expression of p‐eNOS induced by different concentrations of rhVEGF‐C. (B) Level of expression of p‐eNOS in corrected total cell fluorescence units for each group. Scale bar = 20 μm. *P < 0.05; **P < 0.005.

In our previous report, we demonstrated that VEGF‐C can act as a specific upstream regulator of eNOS in the CCA model 11. Therefore, we further explored the role of VEGF‐C in the regulation of eNOS, as well as in the migration and invasion phenotypes of the CCA cell line. We treated the CCA cell line with rhVEGF‐C at different concentrations and observed the level of p‐eNOS (Fig. 1A). The level of p‐eNOS was significantly increased in a dose‐dependent manner when compared with the untreated control (*P* = 0.019, 0.002 and 0.003, respectively). However, a concentration of 200 ng·mL^−1^ of rhVEGF‐C caused a slight decrease in the level of p‐eNOS (Fig. 1B). A concentration of 100 ng·mL^−1^ of rhVEGF‐C caused the highest increase of p‐eNOS in CCA cells.

### rhVEGF‐C induced CCA cell migration

**Figure 2:**
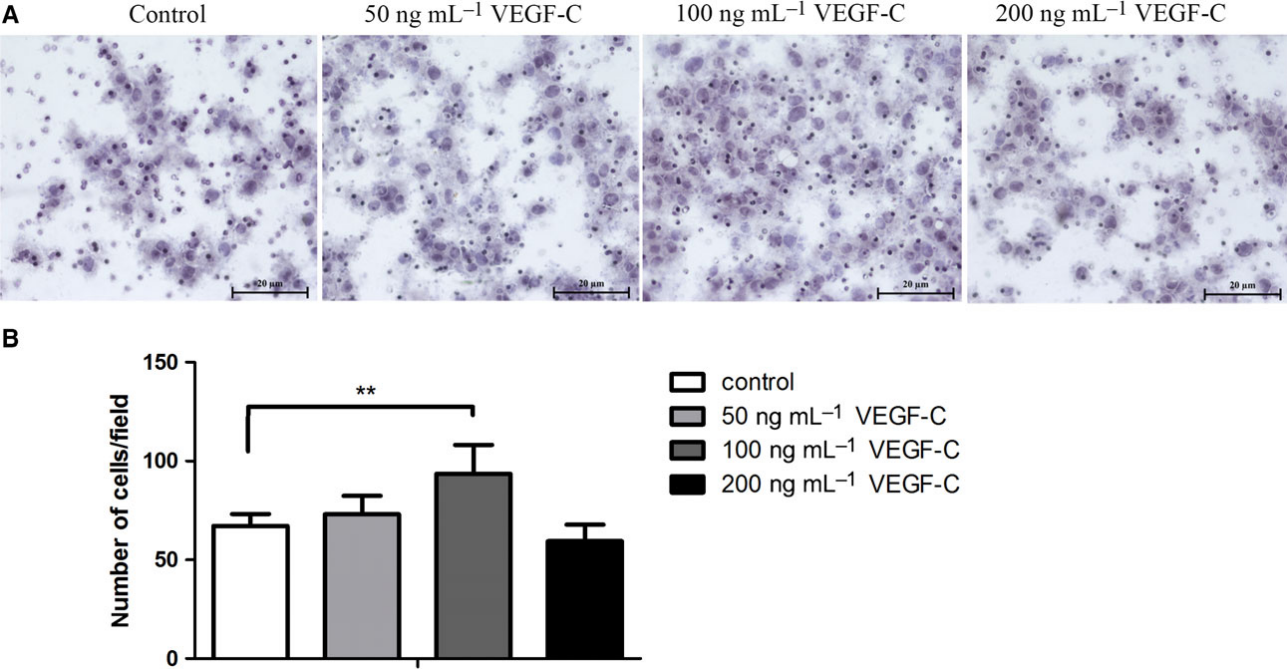
(A) Effect of rhVEGF‐C on CCA cell migration demonstrated using the Boyden chamber transwell assay. (B) Analysis of CCA cell migration (number of migrating cells per field) for each group. Scale bar = 20 μm. **P < 0.005.

Our previous report demonstrated the strong correlation of the expression of eNOS, p‐eNOS and VEGF‐C with the metastatic status of CCA patients 11. Thus we evaluated the migration phenotype of CCA cell lines that were treated with different concentrations of rhVEGF‐C. Interestingly, a significant increase in cell migration was observed in CCA cells that were treated with 100 ng·mL^−1^ of rhVEGF‐C when compared with the untreated control cells (*P* = 0.001) (Fig. 2), whereas migration decreased at 200 ng·mL^−1^, at which concentration a decrease in the level of p‐eNOS was observed.

### rhVEGF‐C induced CCA cell migration through the activation of eNOS

**Figure 3:**
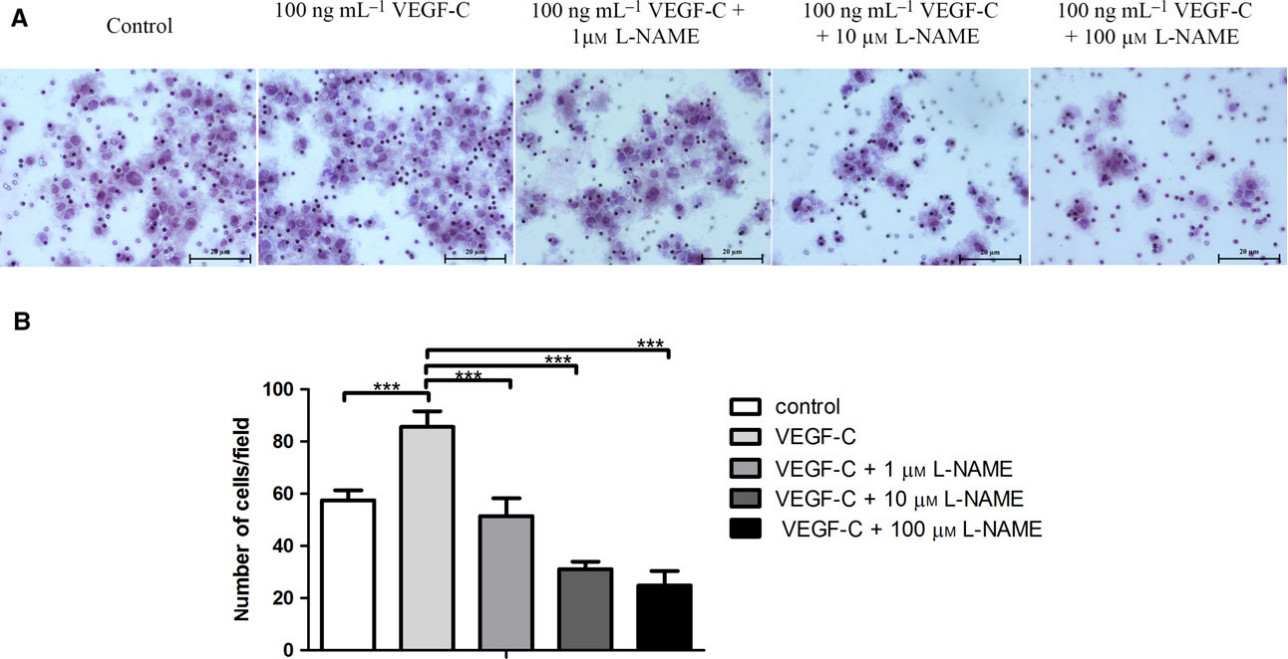
(A) Migration phenotype of the CCA cell line is induced by rhVEGF‐C in combination with rhVEGF‐C and different concentrations of l‐NAME by using the Boyden chamber transwell assay. (B) Analysis of number of migrating cells per field in each experimental group. Scale bar = 20 μm. ***P < 0.0005.

We further explored whether rhVEGF‐C‐induced CCA cell migration was caused, at least in part, by activation of eNOS. We induced CCA cell migration by using rhVEGF‐C in combination with different concentrations of l‐NAME, an eNOS inhibitor, which has no effect on the growth of CCA cell lines (Fig. S1). The result indicated that the migration ability of the CCA cell line is significantly increased when treated with rhVEGF‐C compared with the control (*P* < 0.001). Interestingly, a dramatic decrease in CCA cell migration was seen when l‐NAME was combined in a dose‐dependent manner (*P* < 0.001 in all groups; Fig. 3). Our data confirm that rhVEGF‐C induces CCA cell migration through the activation of eNOS.

### rhVEGF‐C induced CCA cell invasion through the activation of eNOS

**Figure 4:**
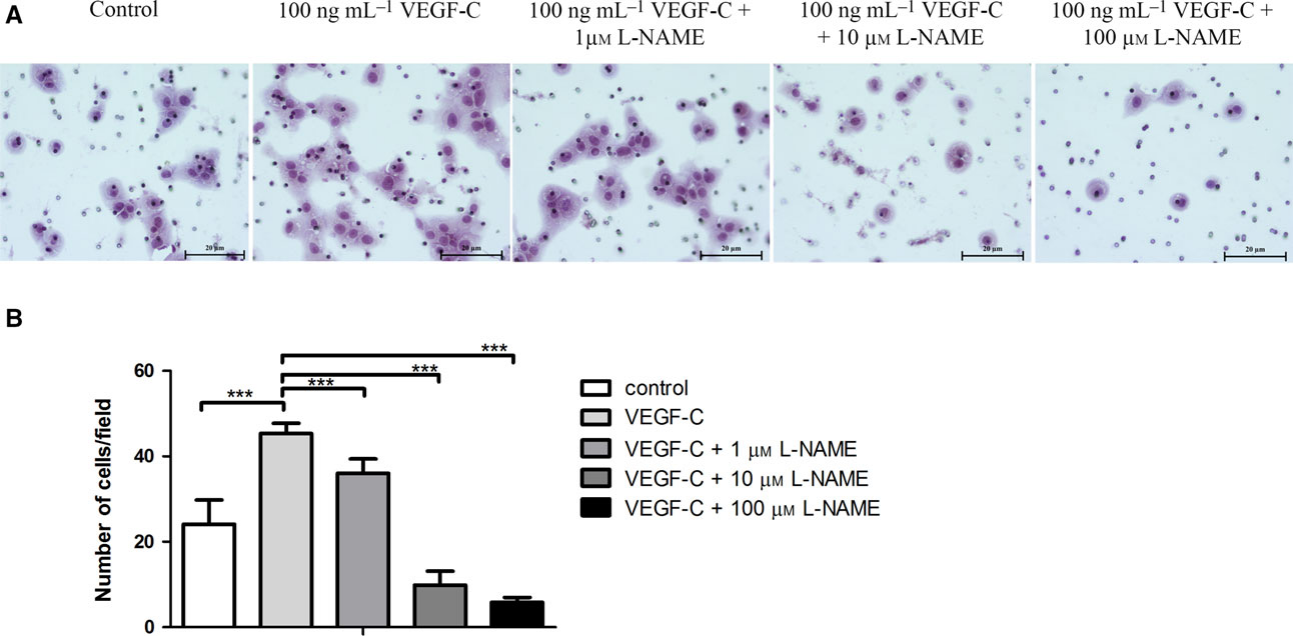
(A) Invasion phenotype of CCA cell line induced by rhVEGF‐C in combination with rhVEGF‐C and different concentrations of l‐NAME. (B) Analysis of number of invading cells per field for each experimental group. Scale bar = 20 μm. ***P < 0.0005.

We also explored the function of eNOS in the invasion by CCA. CCA cells were treated with rhVEGF‐C in combination with different concentrations of l‐NAME after which cell invasion ability was observed. rhVEGF‐C treatment can cause a significant increase in CCA cell invasion when compared with the untreated control cells (*P* < 0.001). On the other hand, the combination of rhVEGF‐C and l‐NAME caused a dramatic decrease of CCA cell invasion in a concentration‐dependent manner (*P* < 0.001 in all groups; Fig. 4). In summary, activation of eNOS is involved in CCA cell invasion.

### l‐NAME suppresses CCA cell migration and invasion *via* an inhibition of Akt, eNOS and VASP activation

**Figure 5:**
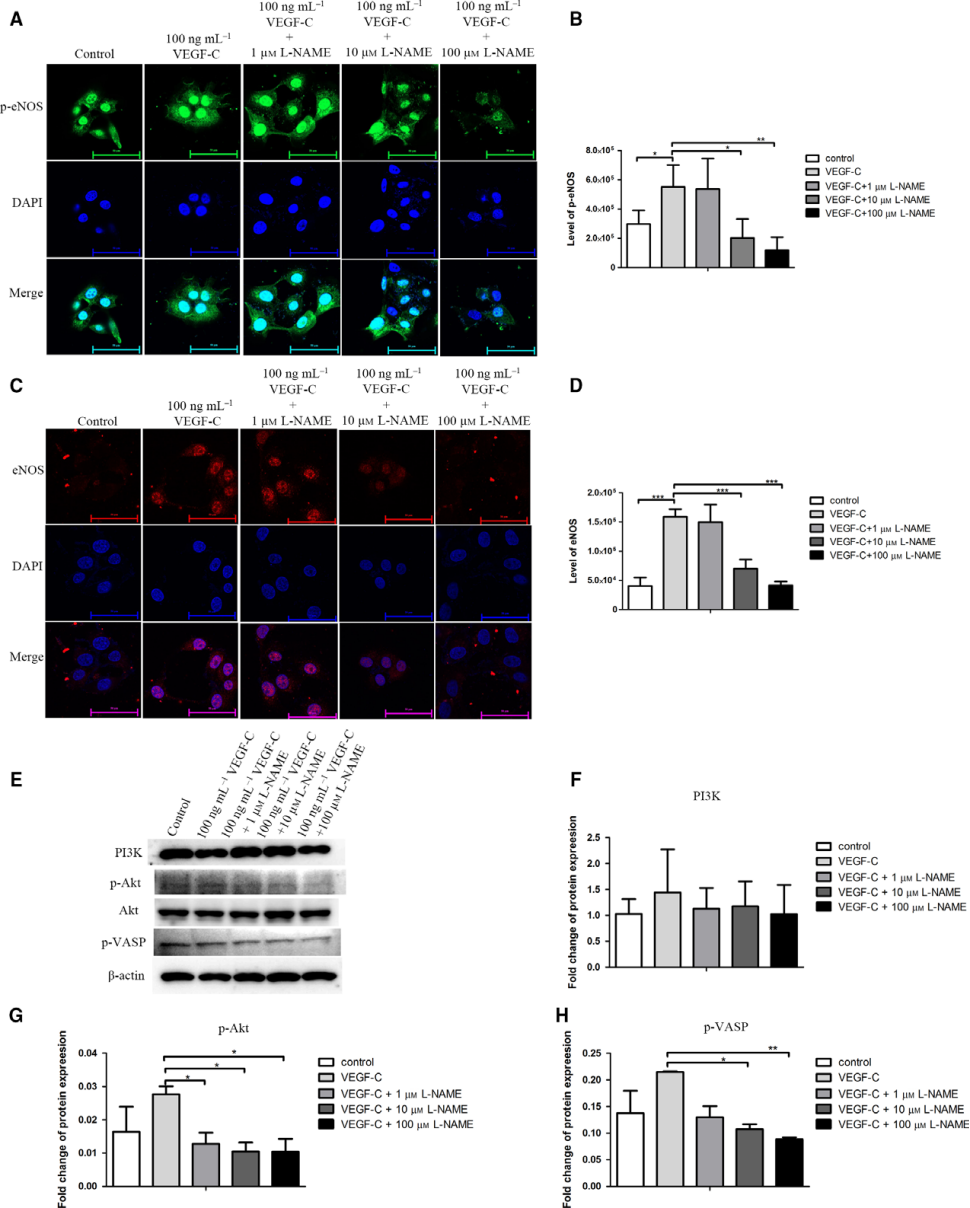
(A) Level of expression of p‐eNOS in each experimental group. DAPI, 4′,6‐diamidino‐2‐phenylindole. (B) Corresponding graph of the level of expression of p‐eNOS. (C,D) Level of expression of eNOS (C) with the corresponding graph (D). (E) Immunoblot of the signaling molecules that are involved in eNOS activation and inhibition. Actin was used as loading control. (F–H) Level of expression of PI3K, p‐Akt and p‐VASP, respectively. Scale bar = 50 μm. *P < 0.05; **P < 0.005; ***P < 0.0005.

Next, we explored the molecular mechanism by which eNOS is involved in CCA progression. Immunolabeling was used to detect the expression p‐eNOS and eNOS (Fig. 5A,C), while immunoblotting was used to detect the expression of the protein kinases PI3K, p‐Akt and Akt. The protein kinase Akt directly phosphorylated eNOS at Ser1177. Then, the VASP protein, which is the downstream effector of eNOS, was activated and phosphorylated at Ser239 (Fig. 5E). The results revealed that rhVEGF‐C treatment significantly increases the expression of eNOS and p‐eNOS (*P* < 0.001 and *P* = 0.035, respectively) (Fig. 5B,D), and could slightly increase the expression of PI3K, p‐Akt and p‐VASP (Fig. 5F–H). In contrast, the combination treatment of rhVEGF‐C with l‐NAME decreased the expression of p‐eNOS, eNOS, p‐Akt and p‐VASP in a concentration‐dependent manner (Fig. 5B,D,G and H, respectively). In particular, the combination of rhVEGF‐C with 10 and 100 μm l‐NAME showed significant decreases in the expression level of p‐eNOS (*P* = 0.013 and *P* = 0.0045 for 10 and 100 μm, respectively). The combination of rhVEGF‐C with 10 and 100 μm l‐NAME also significantly decreased the expression level of eNOS (*P* < 0.001 at both concentrations), as well as p‐VASP (*P* = 0.036 and *P* = 0.0047 for 10 and 100 μm, respectively). The combination treatment of rhVEGF‐C and l‐NAME not only inhibited the expression level of p‐eNOS, eNOS and p‐VASP, but also significantly inhibited the expression level of p‐Akt at all concentrations (*P* = 0.044, 0.022 and 0.049 for 1, 10 and 100 μm l‐NAME, respectively).

Summarizing, eNOS is stimulated *via* the VEGF‐C–PI3K–Akt pathway resulting in an increase in the migration and invasion of the CCA cell line through the up‐regulation of p‐VASP expression. Conversely, the inhibition of eNOS by l‐NAME resulted in a decrease in the migration and invasion abilities of the CCA cell line by suppressing the activation of expression of p‐VASP. The inhibition of eNOS by l‐NAME might act as a negative feedback loop to inhibit the expression level of p‐Akt.

## Discussion

NOS is a nitric oxide generating enzyme with three isoforms: nNOS, iNOS and eNOS. The expression of eNOS occurs mainly in endothelial cells where it promotes the relaxation of vessels through the release of NO 27. However, eNOS is also expressed in several cancer types 18, 20, 28, where it plays a role in cell proliferation, antiapoptosis, angiogenesis, invasion and metastasis 29. The regulation of eNOS is dependent on several upstream regulator proteins 27, 30, 31, among which VEGF‐C and its specific receptor, VEGFR3, have been strongly associated with the expression and activation of eNOS 22.

The alteration of receptor tyrosine kinases and their downstream kinase proteins involved in the development of CCA have been identified. Among the downstream kinases, enhanced expression of eNOS is seen in both CCA cell lines and CCA tissues, with VEGFR3 also being activated 4. The expression of eNOS, p‐eNOS, VEGF‐C and VEGFR3 is significantly associated with metastasis in CCA patients. In addition, the co‐high‐expression of eNOS with its specific upstream regulator proteins, VEGF‐C and VEGFR3, is also significantly associated with metastasis in CCA patients 11. This suggests that eNOS is involved in cancer metastatic processes, especially when VEGF‐C/VEGFR3 is present. This study aimed to explore the function of eNOS in the metastatic process in a CCA cell line upon activation by rhVEGF‐C and inhibition by eNOS inhibitor. Thus, inhibition of eNOS by l‐NAME can serve as a possible strategy to inhibit CCA progression.

Our results show that at a rhVEGF‐C concentration of 100 ng·mL^−1^ there was a significantly increased expression of p‐eNOS and CCA cell migration. In contrast, a high dose of 200 ng·mL^−1^ rhVEGF‐C slightly decreased both the expression of p‐eNOS and CCA cell migration. It is not clear why the reverse effect occurs at high doses of rhVEGF‐C. However, a previous study showed a similar result. In that study, gastric cancer cells were treated with rhVEGF and the concentration measured of interleukin‐18 (IL‐18), which is involved in enhanced cell migration. The rhVEGF increased the concentration of IL‐18 in a dose‐dependent manner, but at 200 ng·mL^−1^ of rhVEGF there was a slight decrease in the IL‐18 concentration. The significant increase in CCA cell migration is consistent with a previous study involving lung cancer cells 32. Interestingly, a combination of rhVEGF‐C and l‐NAME dramatically decreased CCA cell migration ability in a concentration‐dependent manner. That l‐NAME treatment inhibits cancer cell migration has also been found in breast cancer cells in which l‐NAME inhibits 4T1 migration in a dose‐dependent manner 33. It also inhibits the migration of human colorectal cancer cells 34. We demonstrate here for the first time that rhVEGF‐C‐mediated CCA migration is modulated, at least in part, by eNOS activation. In addition, the present study demonstrates that rhVGEF‐C treatment significantly increases CCA cell invasion when compared with the control cells, while l‐NAME inhibited this rhVEGF‐C‐mediated CCA cell invasion ability. Our results are supported by experiments using lung cancer cells, which demonstrated the rhVEGF‐C treatment significantly increased lung cancer cell invasion 32. Moreover, l‐NAME treatment was shown to inhibit human colorectal cancer cell line invasion 34. Although l‐NAME could benefit cancer therapy, the management of the ensuing hypertension, as a side effect, needs to be considered. In a pre‐clinical study, increasing hypertension was observed in an *in vivo* model of pancreatic ductal adenocarcinoma. However, when l‐NAME was provided in combination with amlodipine, an antihypertensive drug, the blood pressure significantly decreased with no effect on the antitumor activity of l‐NAME 17.

Furthermore, the molecular mechanism by which eNOS is involved in CCA cell migration and invasion were explored. The results of immunoblotting and immunolabeling indicated that rhVEGF‐C activates eNOS *via* the PI3K–Akt pathway, which directly phosphorylates eNOS to form p‐eNOS (Ser1177). This conforms with our phosphokinase array result 22, 23, 30. The activation of eNOS increases cell migration and invasion through increasing the expression of p‐VASP (Ser239), downstream of eNOS, which is involved in actin filament formation in cancer cells. The increase of p‐VASP (Ser239) expression has also been reported to play a role in cancer cell migration and invasion in various cancer types 35, 36. Our results, show an increased expression of p‐VASP in conditions of eNOS activation and a decreased expression of p‐VASP in conditions of eNOS inhibition in a dose‐dependent manner. Therefore, the inhibition of eNOS directly affects cancer cell migration and invasion *via* a decrease in the level of p‐VASP (Ser239). Under conditions of l‐NAME treatment, our results indicate that l‐NAME can also suppress the expression of p‐Akt. Similarly, some other studies have reported that the inhibition of eNOS also affects the PI3K–Akt pathway in turn to decrease expression level 37, 38.

## Conclusion

The abundant expression of eNOS and its upstream regulator, the VEGF family, were seen in not only CCA cell lines but also human CCA tissues. Moreover, the abundant expression of eNOS as well as its upstream regulator was also found to be significantly correlated with the metastasis status of CCA patients. However, the function of eNOS‐mediated metastasis of CCA not has been established. Thus, this study presented evidence that showed eNOS induced the migration and invasion of a CCA cell line by the induction of rhVEGF‐C. Interestingly, the inhibition of eNOS by l‐NAME produced a decrease of migration and invasion of cells. The molecular mechanism by which eNOS modulates migration and invasion of cells is through p‐VASP. Therefore, the inhibition of eNOS by l‐NAME might serve as a potentially attractive target to inhibit CCA progression; however, a hypertension side effect would need to be managed.

## Author contributions

MS, AT and WL planned the experiments; MS performed the experiments; MS, AT, NN and PU analyzed the data; TB, AT and PY contributed specimens, reagents or other essential materials; MS and WL wrote the manuscript. All authors read and revised the manuscript.

## Supporting information


**Fig. S1.** Growth inhibitory effect of l‐NAME on CCA cell line, KKU‐213, and immortalized cholangiocyte cell line, MMNK‐1. The cell lines were exposed to l‐NAME at different concentrations of between 0.001 and 100 μm. After 48 h, cell proliferation was detected using the sulforonamide B method. Values of percentage cell growth inhibition are expressed as the mean ± SD of three independent experiments.
