# Supplementary figures and images for: Inhibition of endothelial nitric oxide synthase in cholangiocarcinoma cell lines – a new strategy for therapy
Source: FEBS Open Bio. 2018 Mar 2;8(4):513–22. doi: 10.1002/2211-5463.12388 (PMC5881549; doi:10.1002/2211-5463.12388)

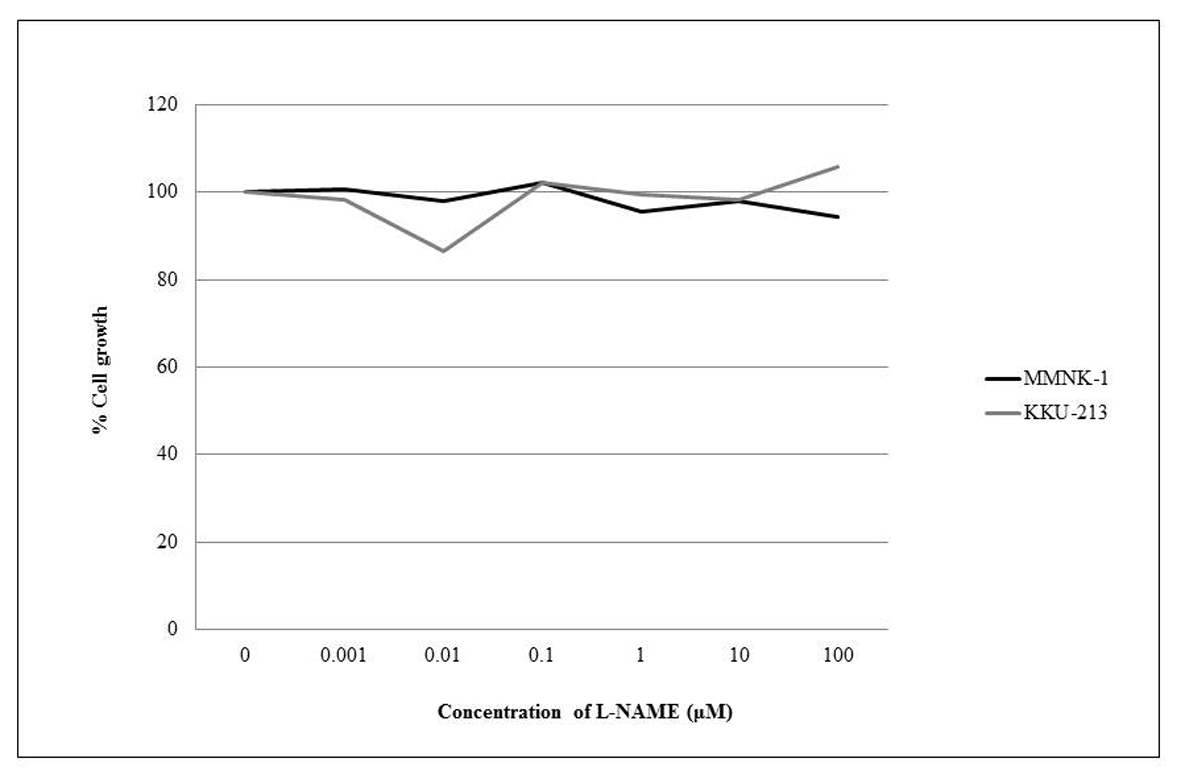

Supplement: Supplementary file 1 — Fig. S1. Growth inhibitory effect of l‐NAME on CCA cell line, KKU‐213, and immortalized cholangiocyte cell line, MMNK‐1. The cell lines were exposed to l‐NAME at different concentrations of between 0.001 and 100 μm. After 48 h, cell proliferation was detected using the sulforonamide B method. Values of percentage cell growth inhibition are expressed as the mean ± SD of three independent experiments. [file FEB4-8-513-s001.tif]
